# Supplementary material for: A high-quality reference genome for the fission yeast Schizosaccharomyces osmophilus
Source: G3 (Bethesda). 2023 Feb 7;13(4):jkad028. doi: 10.1093/g3journal/jkad028 (PMC10085805; doi:10.1093/g3journal/jkad028)
Supplement: jkad028_Supplementary_Data [file jkad028_supplementary_data.zip › Figure_S10_G3-2022-403979.pdf]

Figure S10

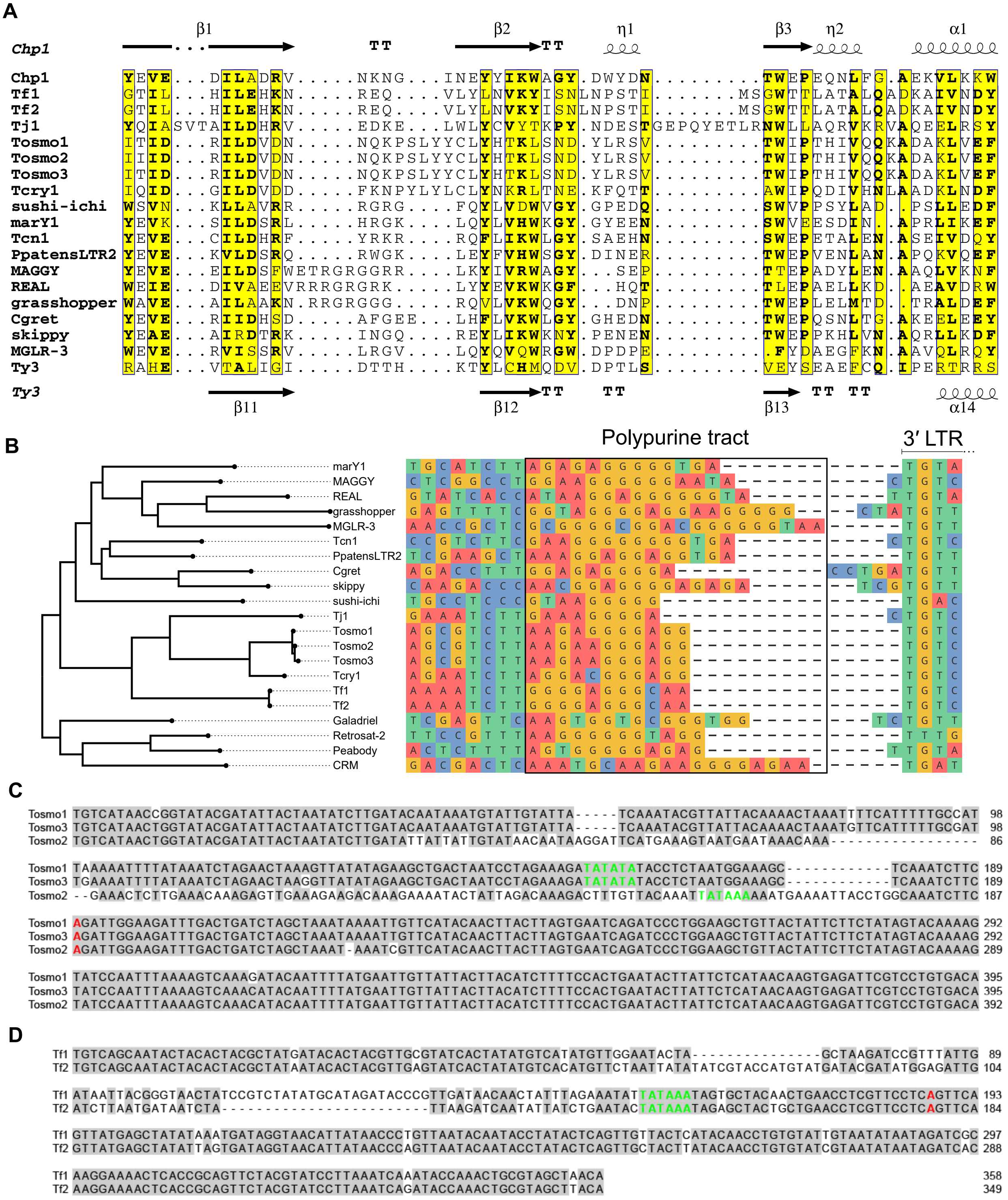

Figure S10. Sequence analyses of chromodomains, PPTs, and LTRs.

(A) Alignment of amino acid sequences of chromodomains. Secondary structures of the chromodomains of *S. pombe* Chp1 (PDB: 3G7L) and Ty3 integrase (PDB: 7Q5B) are shown at top and bottom, respectively.

(B) Alignment of the nucleotide sequences of and surrounding the polypurine tracts (PPTs). The phylogenetic tree shown on the left is the RT-IN tree shown in Figure 4C.

(C) Alignment of the LTR sequences of Tosmo1, Tosmo2, and Tosmo3. Nucleotides identical to the consensus are shaded in gray. Predicted transcription start sites and TATA boxes are highlighted in red and green, respectively. Transcription start sites are at the beginning of the R region and correspond to the 5′ ends of the self-primers shown in Figure 4D.

(D) Alignment of the LTR sequences of Tf1 and Tf2. Nucleotides identical to the consensus are shaded in gray. Transcription start sites and predicted TATA boxes are highlighted in red and green, respectively.
