# Supplementary material for: A high-quality reference genome for the fission yeast Schizosaccharomyces osmophilus
Source: G3 (Bethesda). 2023 Feb 7;13(4):jkad028. doi: 10.1093/g3journal/jkad028 (PMC10085805; doi:10.1093/g3journal/jkad028)
Supplement: jkad028_Supplementary_Data [file jkad028_supplementary_data.zip › Figure_S11_G3-2022-403979.pdf]

Figure S11

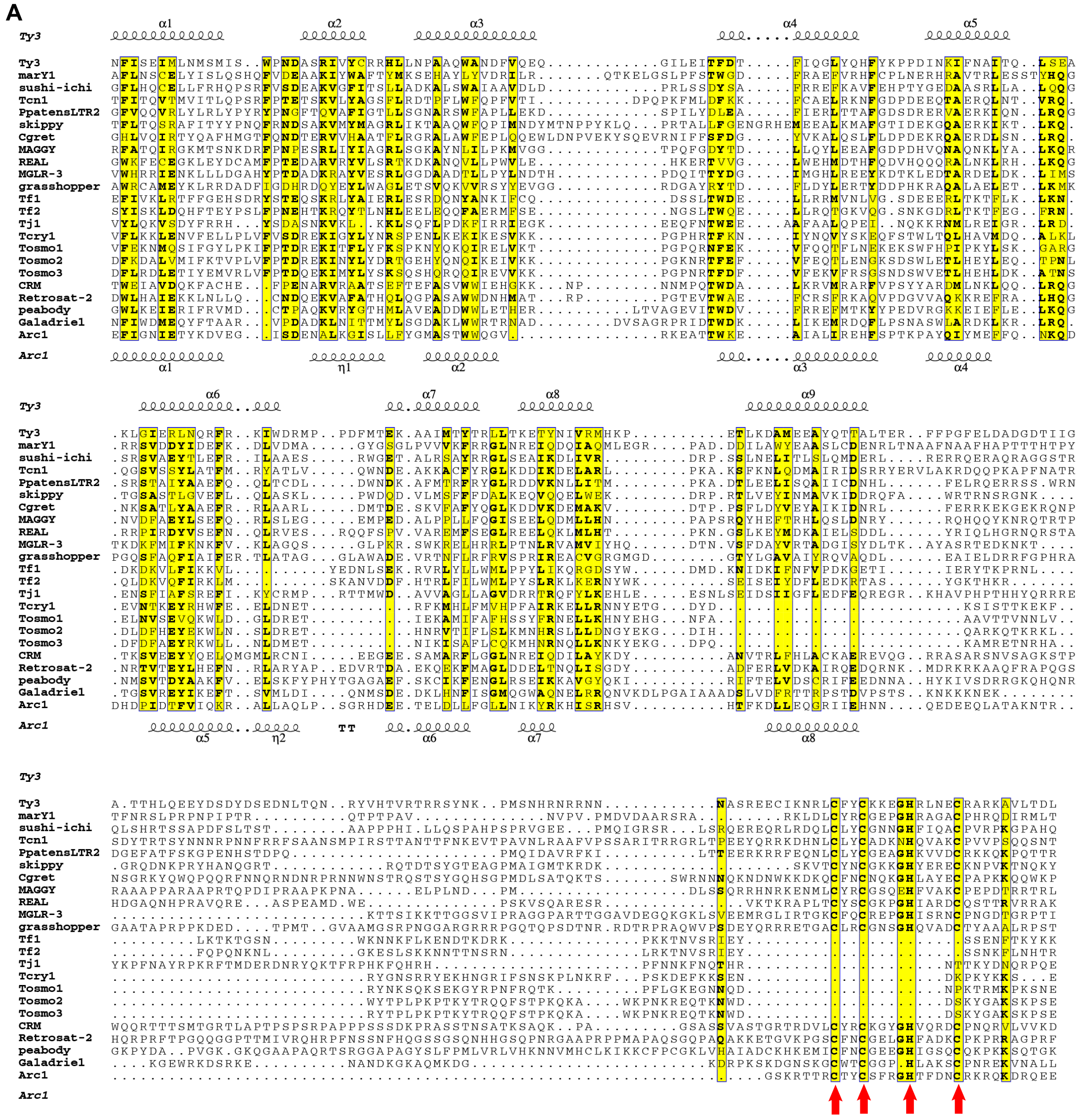

Figure S11. Alignment of amino acid sequences of Gag proteins.

The sequences included in this alignment all begin at a stretch of sequence exhibiting similarity to helix 1 of Ty3 capsid N-terminal domain (CA-NTD). For CCHC-containing Gag proteins, the sequences end at the 10th amino acids downstream of the last cysteine of the CCHC motif; for Tosmo1, Tosmo2, Tosmo3, Tcry1, and Tj1, the sequences end at the last amino acid before the

in-frame stop codon; for Tf1, the sequence ends at amino acid 245 (Teysset *et al.* 2003); for Tf2, the sequence ends at a position homologous to amino acid 245 of Tf1, and the amino acid at this position is the 4th amino acid upstream of the first β strand of the protease fold in the AlphaFold-predicted structure of the protein encoded by Tf2-3, which has a sequence identical to GenBank L10324 (https://alphafold.ebi.ac.uk/entry/P0CT36) (Varadi *et al.* 2022). Secondary structures shown at top are based on the structures of Ty3 CA-NTD (PDB 6R22) and Ty3 CA-CTD (PDB 6R23) (Dodonova *et al.* 2019). The secondary structure shown at bottom are based on the structure of *Drosophila* dArc1 (PDB 6TAS) (Erlendsson *et al.* 2020).
