# Supplementary material for: A high-quality reference genome for the fission yeast Schizosaccharomyces osmophilus
Source: G3 (Bethesda). 2023 Feb 7;13(4):jkad028. doi: 10.1093/g3journal/jkad028 (PMC10085805; doi:10.1093/g3journal/jkad028)
Supplement: jkad028_Supplementary_Data [file jkad028_supplementary_data.zip › Figure_S12_G3-2022-403979.pdf]

Figure S12

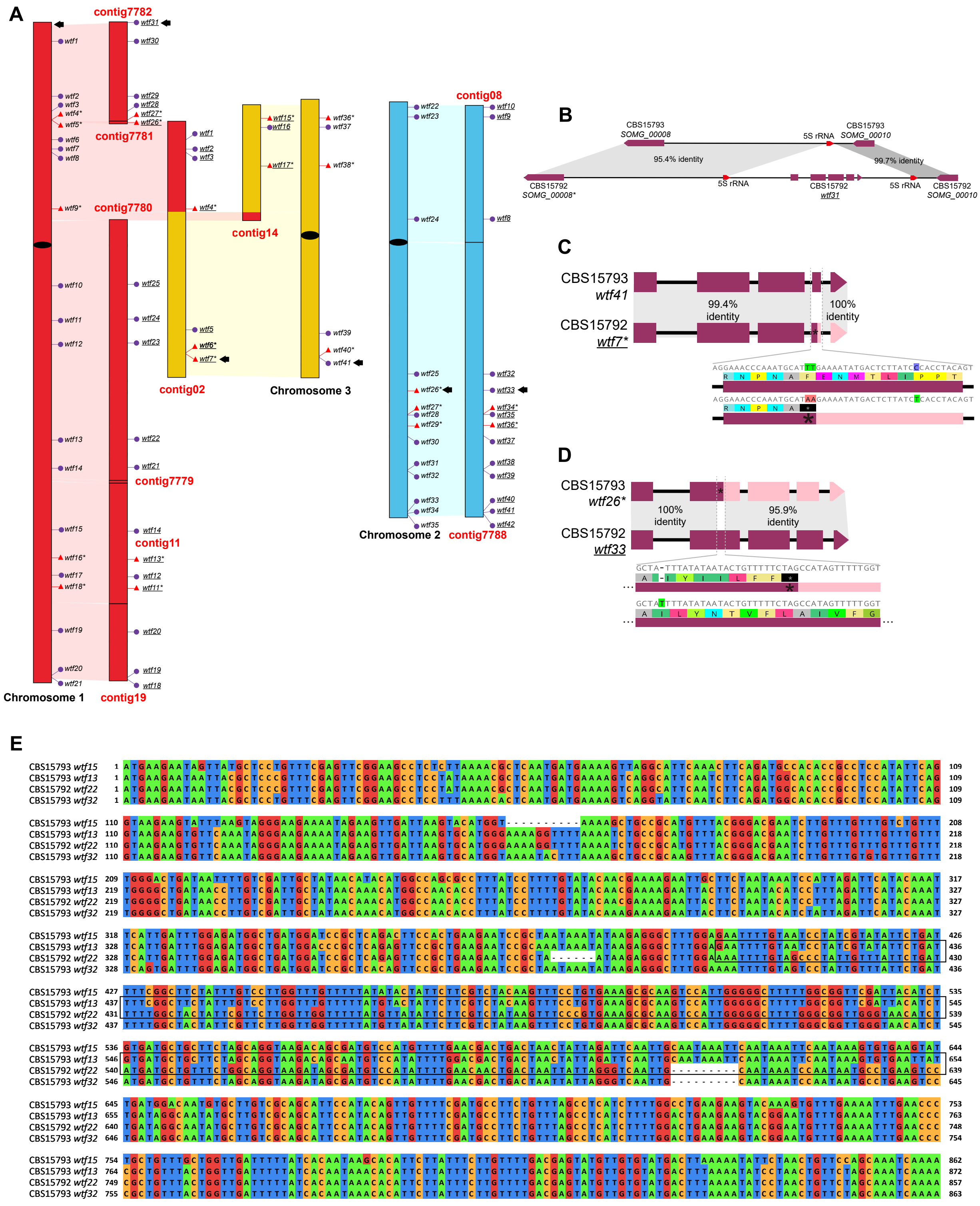

Figure S12. Comparisons of *wtf* genes in CBS 15792 and CBS 15793^{T}.

(A) The genomic distribution of *wtf* genes in CBS 15793^{T} and CBS 15792. The synteny between the three chromosomes of CBS 15793^{T} and the 10 nuclear contigs of CBS 15972 is indicated by colored connections (red, blue, and yellow for chromosomes 1, 2, and 3 of CBS 15793^{T}, respectively). Centromeres in the chromosomes of CBS 15793^{T} are denoted by black ovals. The names of the 10 contigs of CBS 15972 are abbreviated such as the contig tig00007780_pilon_x4, for example, is written as contig7780. Active genes and pseudogenes are denoted by purple dots and red triangles, respectively. The 41 *wtf* genes of CBS 15793^{T} are named according to their positions in the genome. The names of the 42 *wtf* genes of CBS 15792, which are underlined, are as described (De Carvalho *et al.* 2022). The names of pseudogenes are marked with an asterisk. The presence–absence polymorphism shown in B and the differences in the status as an active gene or a pseudogene shown in C and D are highlighted using black arrows.

(B) Schematic illustrating the presence–absence polymorphism of the *wtf31* gene of CBS 15792.

(C) Schematic illustrating the sequence difference that results in *wtf7* of CBS 15792 being a pseudogene but its syntenic counterpart *wtf41* of CBS 15793^{T} being an active gene.

(D) Schematic illustrating the sequence difference that results in *wtf33* of CBS 15792 being an active gene and its syntenic counterpart *wtf26* of CBS 15793^{T} being a pseudogene.

(E) Multiple sequence alignment illustrating the sequence divergence between *wtf13* in CBS 15793^{T} and its syntenic counterpart *wtf22* in CBS 15792. Black box highlights the approximately 250-bp region where ectopic recombination may have occurred and resulted in a low nucleotide identity of 85.36% between these two syntenic genes.
