# Supplementary material for: A high-quality reference genome for the fission yeast Schizosaccharomyces osmophilus
Source: G3 (Bethesda). 2023 Feb 7;13(4):jkad028. doi: 10.1093/g3journal/jkad028 (PMC10085805; doi:10.1093/g3journal/jkad028)
Supplement: jkad028_Supplementary_Data [file jkad028_supplementary_data.zip › Figure_S13_G3-2022-403979.pdf]

Figure S13

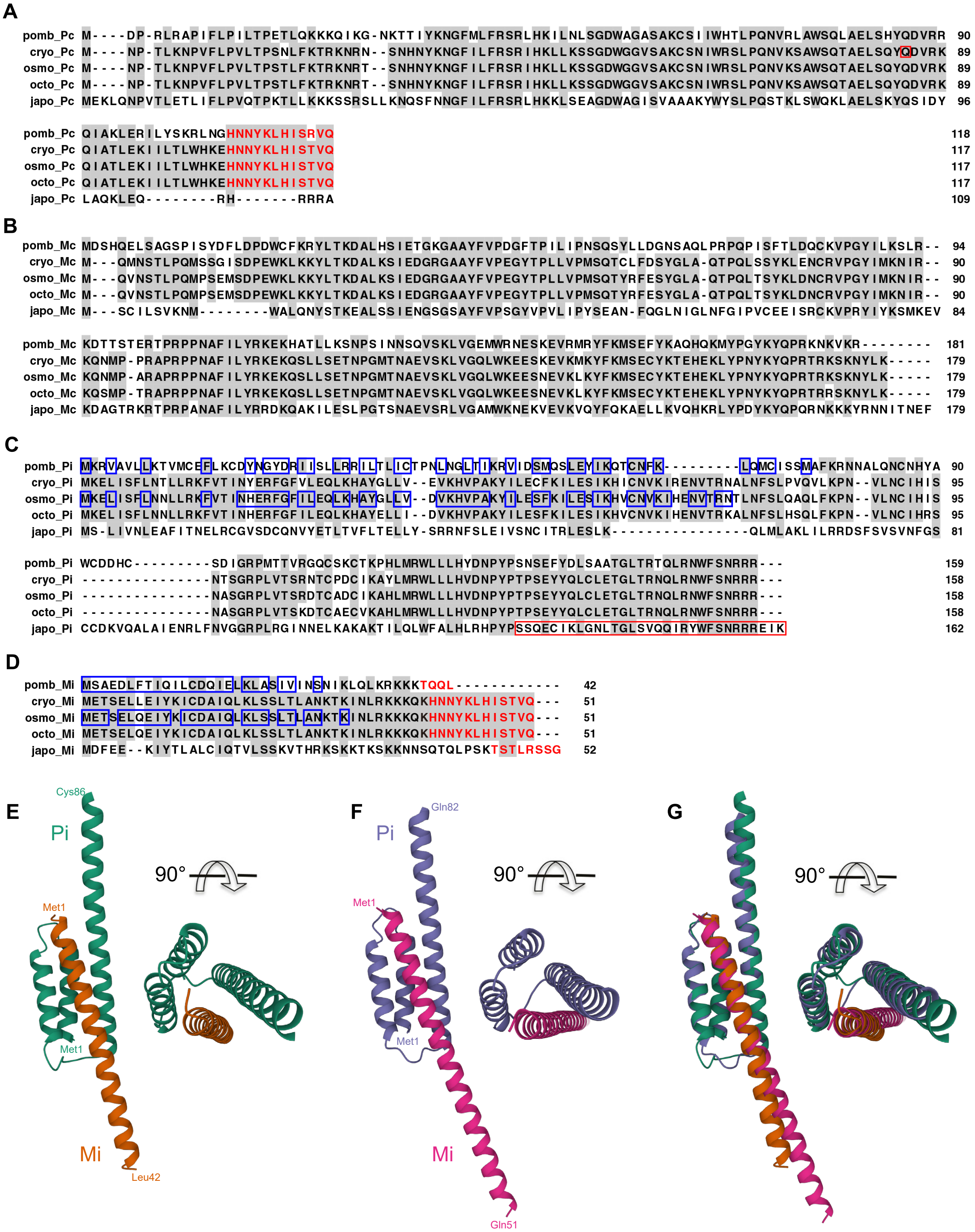

Figure S13. Sequences and predicted structures of mating-type cassette-encoded proteins.

(A–D) Multiple sequence alignments of Pc (A), Mc (B), Pi (C), and Mi (D) of five fission yeast species. Residues identical to the consensus are shaded in gray. Residues encoded by sequences in the H2 boxes are highlighted in red letters. The Pc residue where a premature stop codon occurs in the *S. cryophilus* genome assembly reported in Tong et al. 2019 is highlighted by a red square box. The Pi residues affected by a frameshifting 1-bp deletion present in the reference *S. japonicus* genome are highlighted by a red rectangle box. Pi and Mi residues situated at the intermolecular interfaces of the AlphaFold-Multimer-predicted Pi–Mi heterodimer structures are highlighted by blue boxes.

(E–F) AlphaFold-Multimer-predicted heterodimeric structures of the Pi–Mi complex of *S. pombe* (E) and the Pi–Mi complex of *S. osmophilus* (F). For clarity, only the N-terminal Mi-interacting region of Pi is shown.

(G) Superposition of the structures shown in (E) and (F). The two structures were superposed using residues 1–67 of *S. pombe* Pi and residues 1–65 of *S. osmophilus* Pi.
