# Supplementary material for: A high-quality reference genome for the fission yeast Schizosaccharomyces osmophilus
Source: G3 (Bethesda). 2023 Feb 7;13(4):jkad028. doi: 10.1093/g3journal/jkad028 (PMC10085805; doi:10.1093/g3journal/jkad028)
Supplement: jkad028_Supplementary_Data [file jkad028_supplementary_data.zip › Figure_S14_G3-2022-403979.pdf]

Figure S14

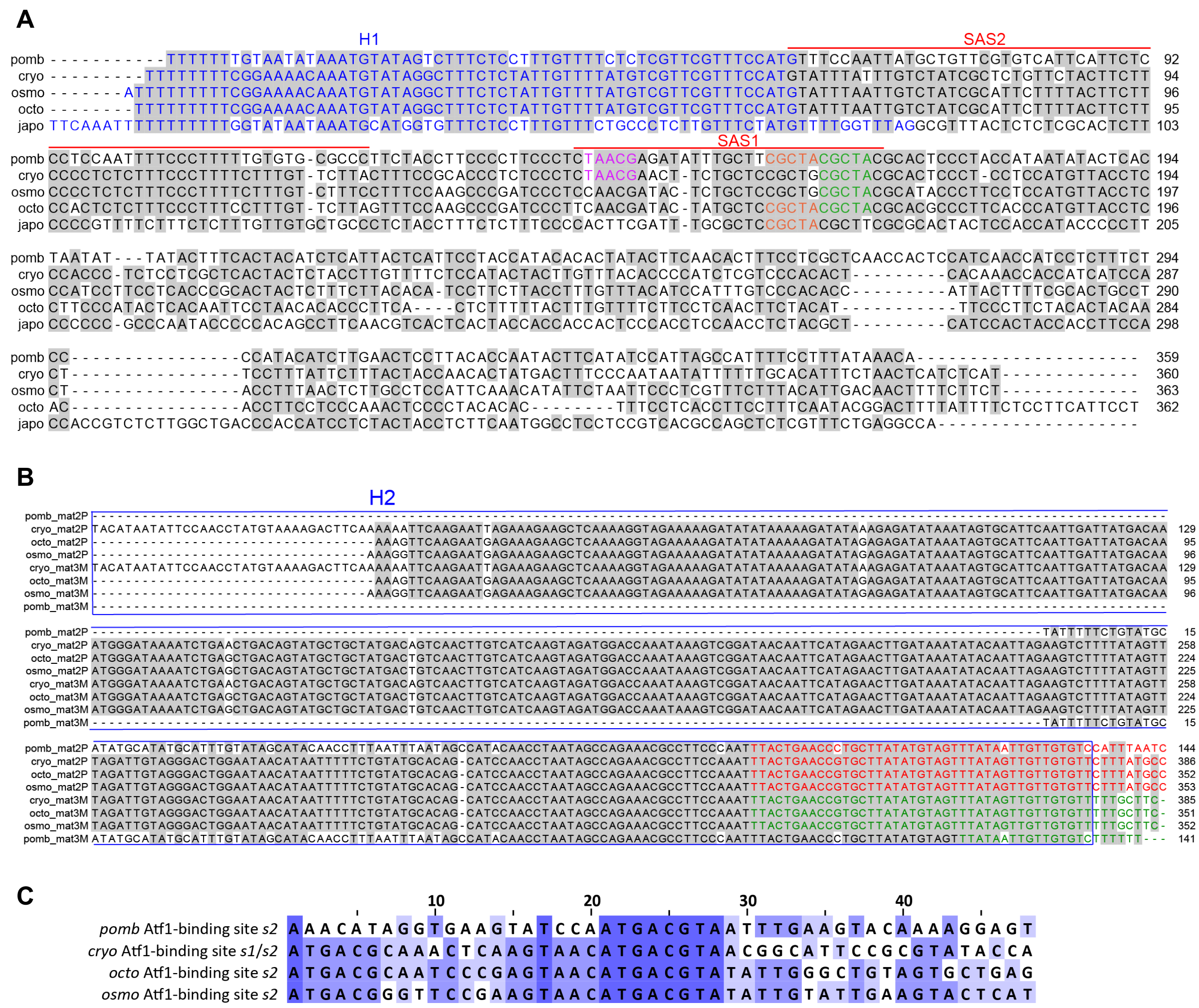

Figure S14. Conservation of H1, H2, the *L*-region segment containing SAS1 and SAS2, and the Atf1-binding sites.

(A) Alignment of the nucleotide sequences of the 300-bp region that begins from the cassette-proximal border of H1 and extends into the *L*-region. Nucleotides identical to the consensus are shaded in gray. H1 sequences are highlighted in blue letters. SAS1 and SAS2 of *S. pombe* are highlighted by horizontal lines. Three TA(A/G)CG motifs in SAS1 of *S. pombe* are highlighted in pink, orange, and green letters. TA(A/G)CG motifs in

SAS1 counterparts in *S. cryophilus*, *S. osmophilus*, *S. octosporus*, and *S. japonicus* are highlighted similarly.

(B) Alignment of the nucleotide sequences of H2 boxes of *S. pombe*, *S. octosporus*, *S. cryophilus*, and *S. osmophilus*. Nucleotides identical to the consensus are shaded in gray. Coding sequences for Pc and Mi are highlighted in red letters and green letters, respectively.

(C) The 48-bp sequences centered on the most cassette-proximal 8-bp Atf1-binding sites (ATGACGTA) in the donor regions of *S. pombe*, *S. cryophilus*, *S. octosporus*, and *S. osmophilus*. The *s1* and *s2* sites of *S. cryophilus* are located within the H3 boxes and therefore share the same flanking sequences. The *s2* site of *S. octosporus* is 33 bp away from the nearby H3 box and the *s2* site of *S. osmophilus* is 30 bp away from the nearby H3 box. Sequence shading is based on the level of sequence identity.
