# Supplementary material for: A high-quality reference genome for the fission yeast Schizosaccharomyces osmophilus
Source: G3 (Bethesda). 2023 Feb 7;13(4):jkad028. doi: 10.1093/g3journal/jkad028 (PMC10085805; doi:10.1093/g3journal/jkad028)
Supplement: jkad028_Supplementary_Data [file jkad028_supplementary_data.zip › Figure_S15_G3-2022-403979.pdf]

Figure S15

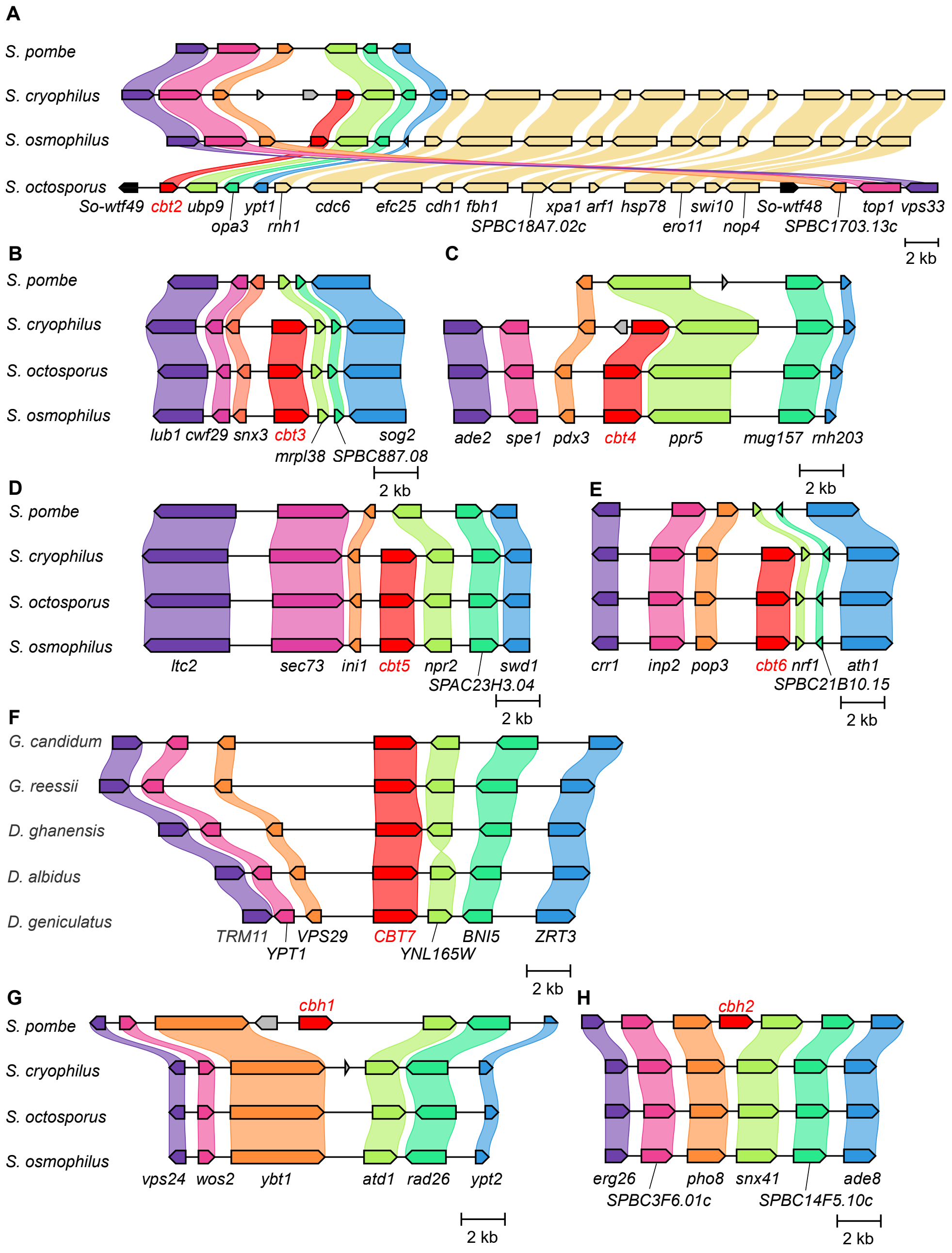

Figure S15. Diagrams showing the local synteny at genomic regions containing genes encoding Cbp1 family proteins, including Cbt2 (A), Cbt3 (B), Cbt4 (C), Cbt5 (D), Cbt6 (E), Cbt7 (F), Cbh1 (G), and Cbh2 (H). In (F), except for *CBT7*, gene names shown are the names of the homologous genes in *Saccharomyces cerevisiae*. In the other panels, except for *cbt1*–*cbt6* and two *wtf* genes in *S. octopous* (*wtf48*/*SOCG_00278* and *wtf49*/*SOCG_00295*), gene names shown are the names of the genes in *S. pombe*.
