# Supplementary material for: A high-quality reference genome for the fission yeast Schizosaccharomyces osmophilus
Source: G3 (Bethesda). 2023 Feb 7;13(4):jkad028. doi: 10.1093/g3journal/jkad028 (PMC10085805; doi:10.1093/g3journal/jkad028)
Supplement: jkad028_Supplementary_Data [file jkad028_supplementary_data.zip › Figure_S16_G3-2022-403979.pdf]

Figure S16

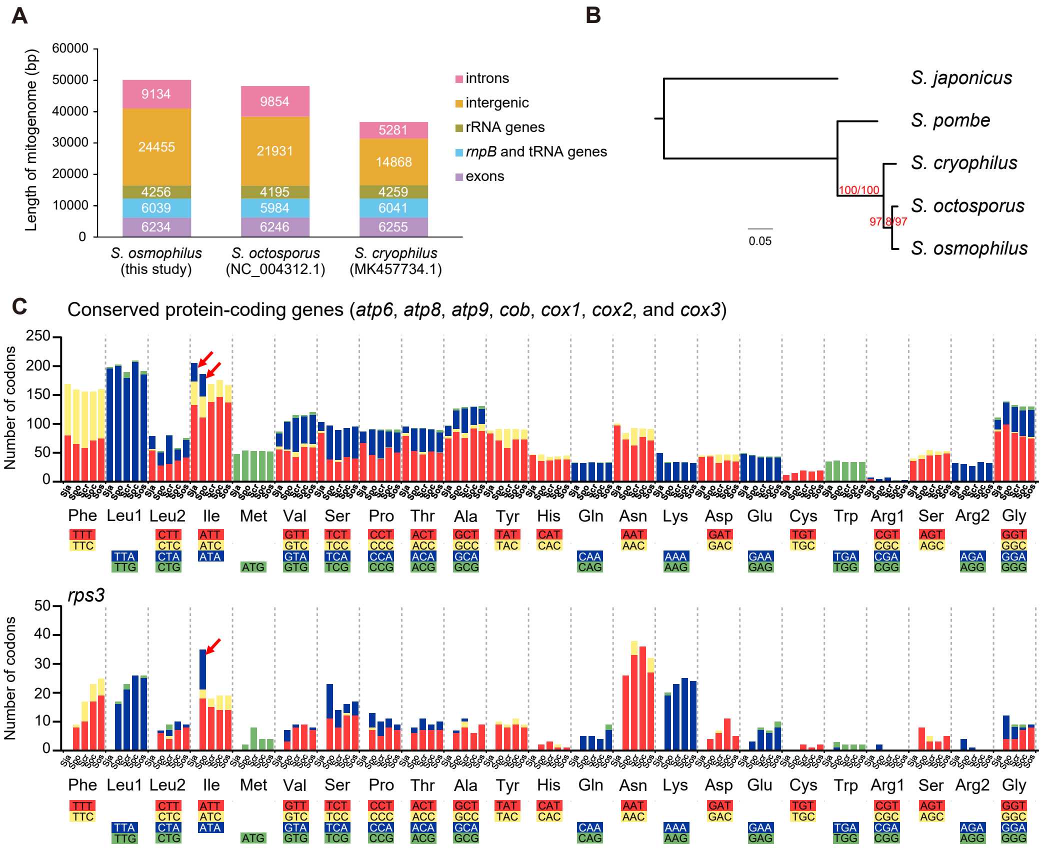

D

	cox1								*cox2*	*cob*	
	S206	P386	P720	P731	P951	S961	P1107	S1138	S373	P429	S687
S. japonicus			IB (complete)	IB (complete)							
S. pombe	II (domainV)	I (derived, B1)	I (derived, B1)	IB (complete)			I (derived, B2)	II (domainV)	II (domainV)		II (domainV)
S. cryophilus		IB (complete)	IB (complete)	IB (complete)			I (derived, B2)			ID	
S. octosporus		IB (complete)	IB (complete)	IB (complete)		II (domainV)	I (derived, B2)		II (domainV)		
S. osmophilus	II (domainV)	IB (complete)		IB (complete)	ID		I (derived, B2)	II (domainV)			

with IEP

without IEP

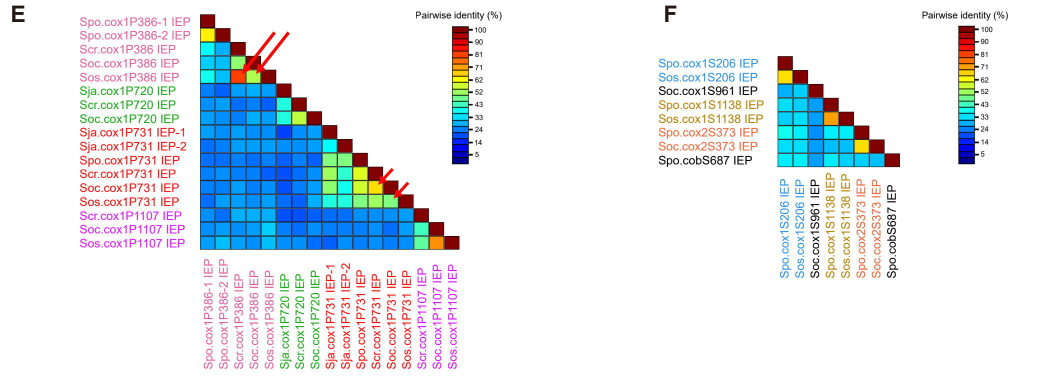

Figure S16. Comparative analysis of the mitogenomes of five fission yeast species.

(A) Length differences between the mitogenomes of *S. octosporus*, *S. osmophilus*, and *S. cryophilus* are mainly due to variations in the lengths of introns and intergenic regions.

(B) Maximum likelihood tree constructed using the amino acid sequences of the proteins encoded by the seven conserved protein-coding genes (*atp6*, *atp8*, *atp9*, *cob*, *cox1*, *cox2*, and *cox3*) in the fission yeast mitogenomes. The tree was rooted using *S. japonicus* as outgroup. Branch labels are the SH-aLRT support value (%) and the UFBoot support value (%) calculated by IQ-TREE.

(C) Codon usage in the mitogenomes of five fission yeast species. Red arrows highlight the presence of ATA codons in *S. japonicus* and *S. pombe*. Sja, *S. japonicus*; Spo, *S. pombes*; Scr, *S. cryophilus*; Soc, *S. octosporus*; Sos, *S. osmophilus*.

(D) Mitochondrial introns of five fission yeast species. The *S. pombe* introns listed include all introns known to exist in natural isolates of *S. pombe* (Tao *et al.* 2019). The introns of the other four species are introns present in the reference strains. RNAweasel-annotated intron type (group I or group II) and subgroup classification for group I introns are shown. Yellow and blue backgrounds indicate the presence and absence of intron-encoded proteins (IEPs), respectively. *S. pombe* has two different cox1P386 introns (cox1P386-1 and cox1P386-2). They belong to the same subgroup and both encode IEPs.

(E) A color matrix showing the pair-wise amino acid identities between proteins encoded by group I introns. This color matrix was generated using Sequence Demarcation Tool Version 1.2 (SDTv1.2) (Muhire *et al.* 2014). The two long arrows highlight that the identity between Sos.cox1P386 IEP and Scr.cox1P386 IEP was higher than the identity between Sos.cox1P386 IEP and Soc.cox1P386 IEP. The two short arrows highlight that the identity between Soc.cox1P731 IEP and Scr.cox1P731 IEP was higher than the identity between Sos.cox1P731 IEP and Soc.cox1P731 IEP.

(F) A color matrix showing the pair-wise amino acid identities between proteins encoded by group II introns. This color matrix was generated using Sequence Demarcation Tool Version 1.2 (SDTv1.2) (Muhire *et al.* 2014).
