# Supplementary material for: A high-quality reference genome for the fission yeast Schizosaccharomyces osmophilus
Source: G3 (Bethesda). 2023 Feb 7;13(4):jkad028. doi: 10.1093/g3journal/jkad028 (PMC10085805; doi:10.1093/g3journal/jkad028)
Supplement: jkad028_Supplementary_Data [file jkad028_supplementary_data.zip › Figure_S17_G3-2022-403979.pdf]

Figure S17

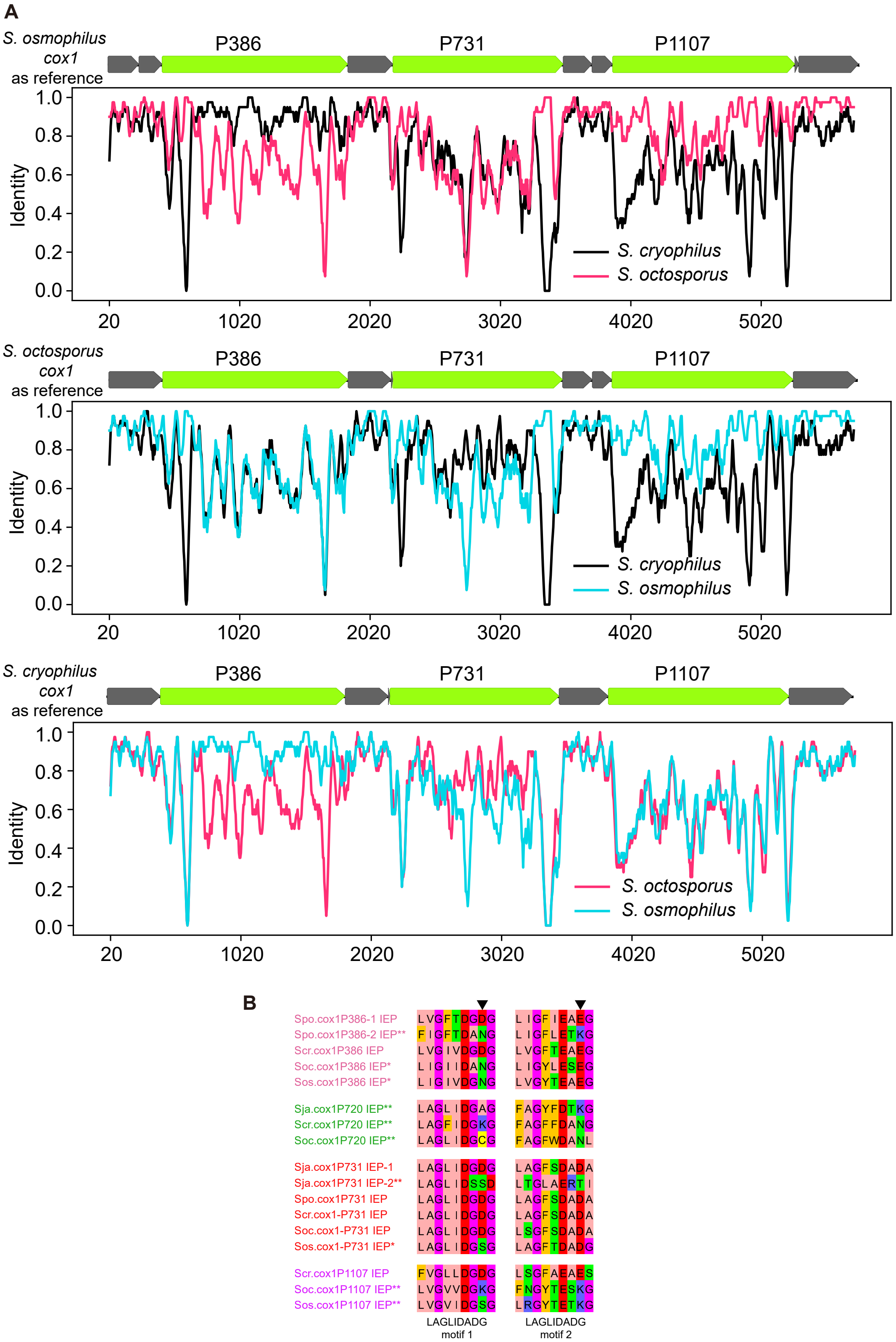

Figure S17. Comparative analysis of the mitogenomes of five fission yeast species.

(A) Sliding-window analysis using SimPlot++ showed that the sequences that may have undergone introgression or horizontal transfer were limited within the cox1P386 intron and the cox1P731 intron. Introns that are not present in all three mitogenomes were removed before performing sequence alignment and SimPlot++ analysis.

(B) The two LAGLIDADG motifs in the proteins encoded by group I introns. Black arrowheads point to the two catalytic residues, which must both be acidic residues to support DNA cleavage activities. Single asterisks indicate that the catalytic residue in one of the two LAGLIDADG motifs is not an acidic residue. Double asterisks indicate that the catalytic residue in neither LAGLIDADG motif is an acidic residue. Sja.cox1P731 intron encodes two IEPs (IEP-1 and IEP-2) and only one of them is degenerated.
