# Supplementary material for: A high-quality reference genome for the fission yeast Schizosaccharomyces osmophilus
Source: G3 (Bethesda). 2023 Feb 7;13(4):jkad028. doi: 10.1093/g3journal/jkad028 (PMC10085805; doi:10.1093/g3journal/jkad028)
Supplement: jkad028_Supplementary_Data [file jkad028_supplementary_data.zip › Figure_S1_G3-2022-403979.pdf]

Figure S1

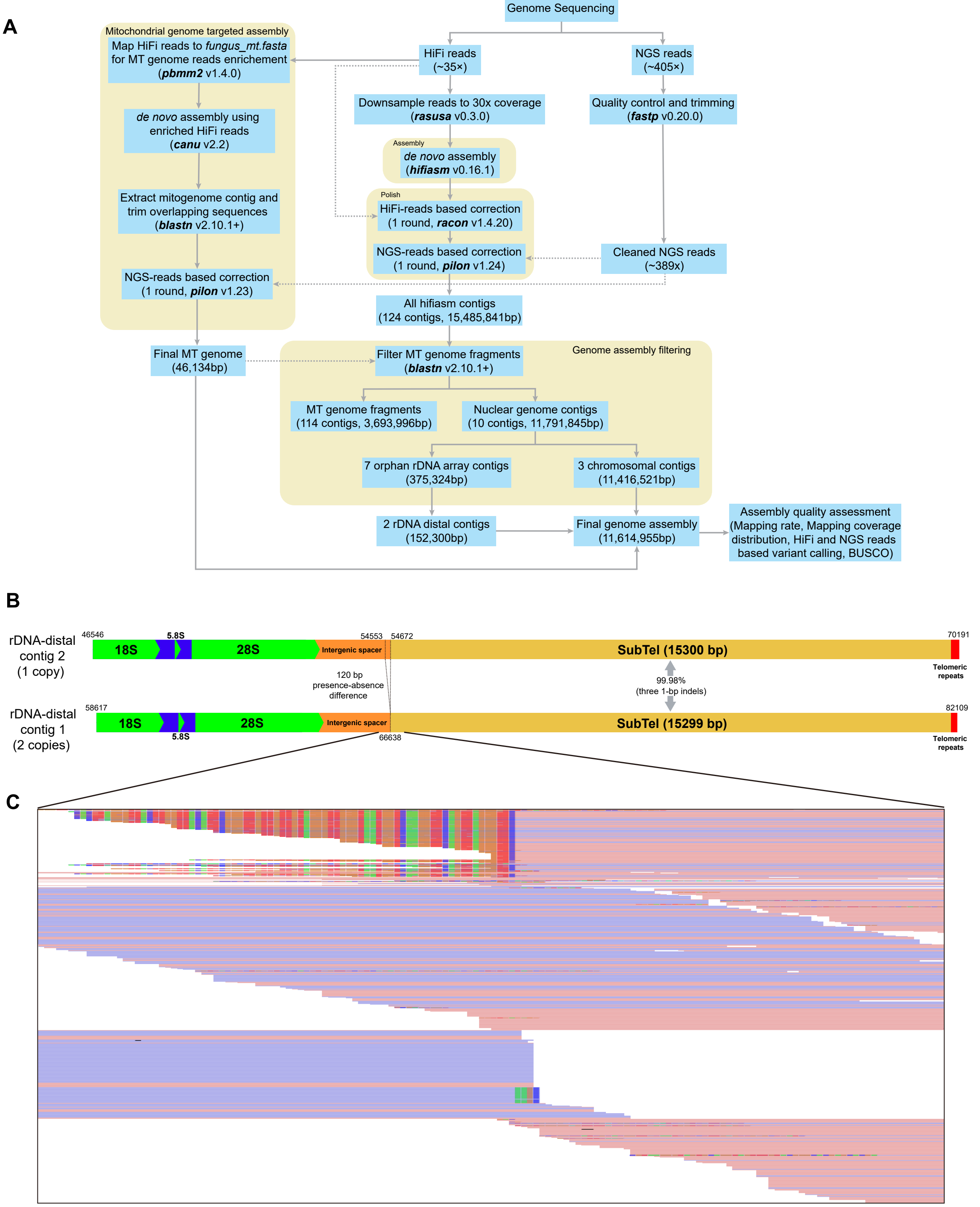

Figure S1. Assembling the genome of the *S. osmophilus* type strain CBS 15793^{T}.

(A) Workflow of genome assembly.

(B) Diagrams of the two rDNA-distal contigs.

(C) IGV screenshot showing the result of mapping all paired-end Illumina reads to the rDNA-distal contig-1. Reads that show perfect mapping at the rDNA/non-rDNA junction are about twice as many as reads that are soft-clipped at the rDNA/non-rDNA junction. The soft-clipped portions match the sequence at the rDNA/non-rDNA junction of the rDNA-distal contig-2.
