# Supplementary material for: A high-quality reference genome for the fission yeast Schizosaccharomyces osmophilus
Source: G3 (Bethesda). 2023 Feb 7;13(4):jkad028. doi: 10.1093/g3journal/jkad028 (PMC10085805; doi:10.1093/g3journal/jkad028)
Supplement: jkad028_Supplementary_Data [file jkad028_supplementary_data.zip › Figure_S2_G3-2022-403979.pdf]

Figure S2

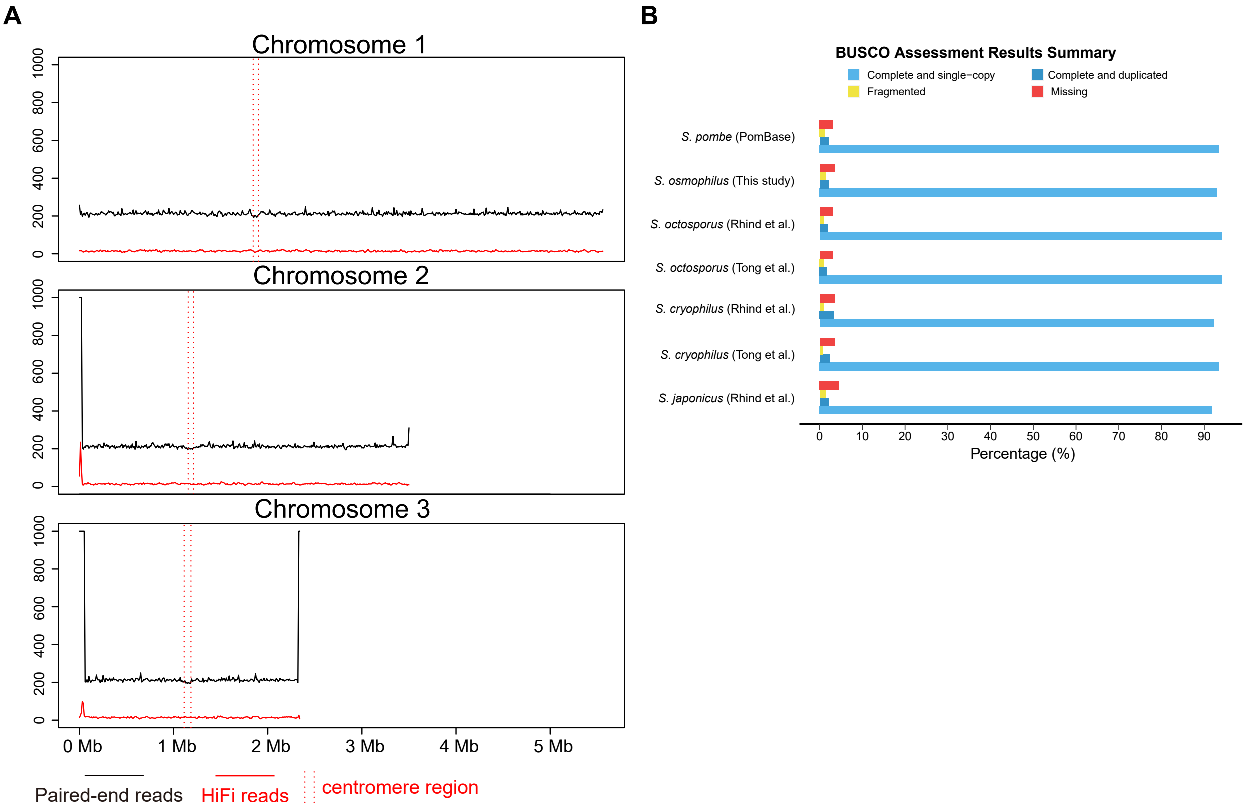

Figure S2. Quality assessment of the genome assembly.

(A) Sliding-window analysis (10-kb window) of the depth coverage of PacBio HiFi reads (red line) and Illumina paired-end reads (black line) along the chromosomal contigs.

(B) BUSCO completeness assessment of the *S. osmophilus* genome assembly and six published genomes of *S. pombe*, *S. octosporus*, *S. cryophilus*, and *S. japonicus*.
