# Supplementary material for: A high-quality reference genome for the fission yeast Schizosaccharomyces osmophilus
Source: G3 (Bethesda). 2023 Feb 7;13(4):jkad028. doi: 10.1093/g3journal/jkad028 (PMC10085805; doi:10.1093/g3journal/jkad028)
Supplement: jkad028_Supplementary_Data [file jkad028_supplementary_data.zip › Figure_S3_G3-2022-403979.pdf]

Figure S3

A

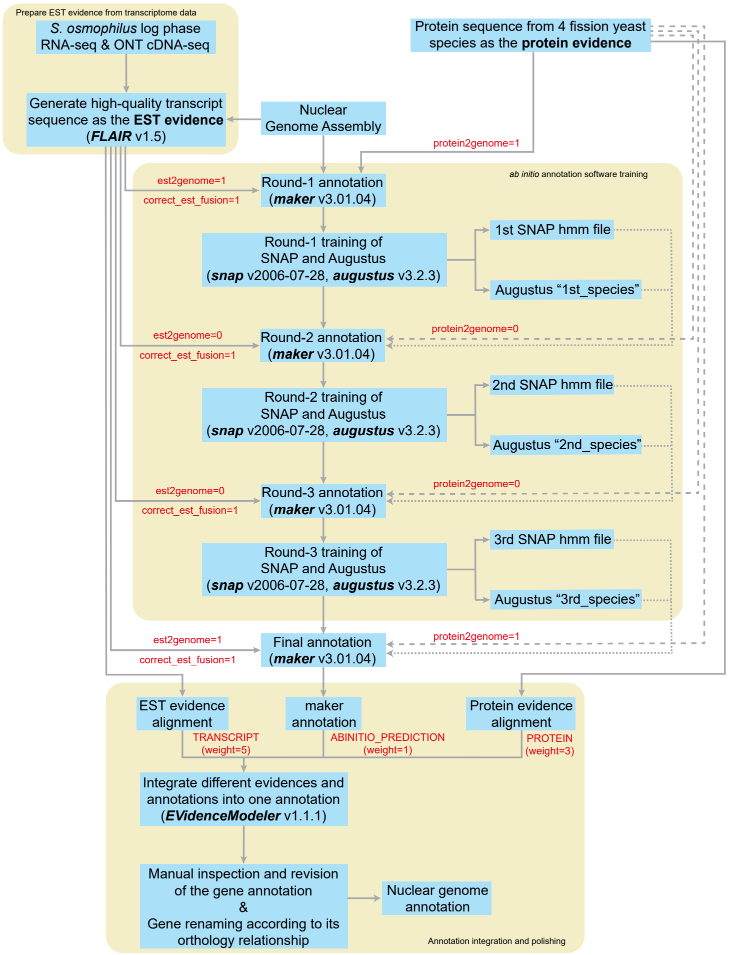

B

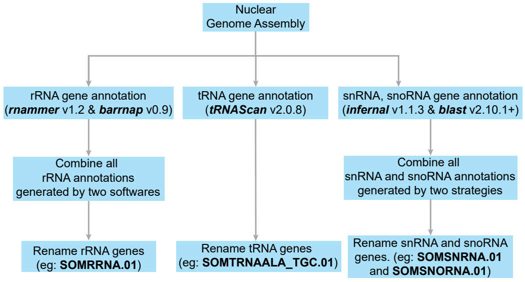

C

Type	Number of copies
Protein coding genes	5098
tRNA genes	293
5S rRNA genes	102
snRNA genes	6
snoRNA genes	55

Figure S3. Annotating the genes in the genome assembly of the *S. osmophilus* type strain CBS 15793^{T}.

(A) Workflow of annotating protein-coding genes in the nuclear genome.

(B) Workflow of annotating non-coding RNA genes in the nuclear genome.

(C) Summary of the annotated genes in the nuclear genome .
