# Supplementary material for: A high-quality reference genome for the fission yeast Schizosaccharomyces osmophilus
Source: G3 (Bethesda). 2023 Feb 7;13(4):jkad028. doi: 10.1093/g3journal/jkad028 (PMC10085805; doi:10.1093/g3journal/jkad028)
Supplement: jkad028_Supplementary_Data [file jkad028_supplementary_data.zip › Figure_S4_G3-2022-403979.pdf]

Figure S4

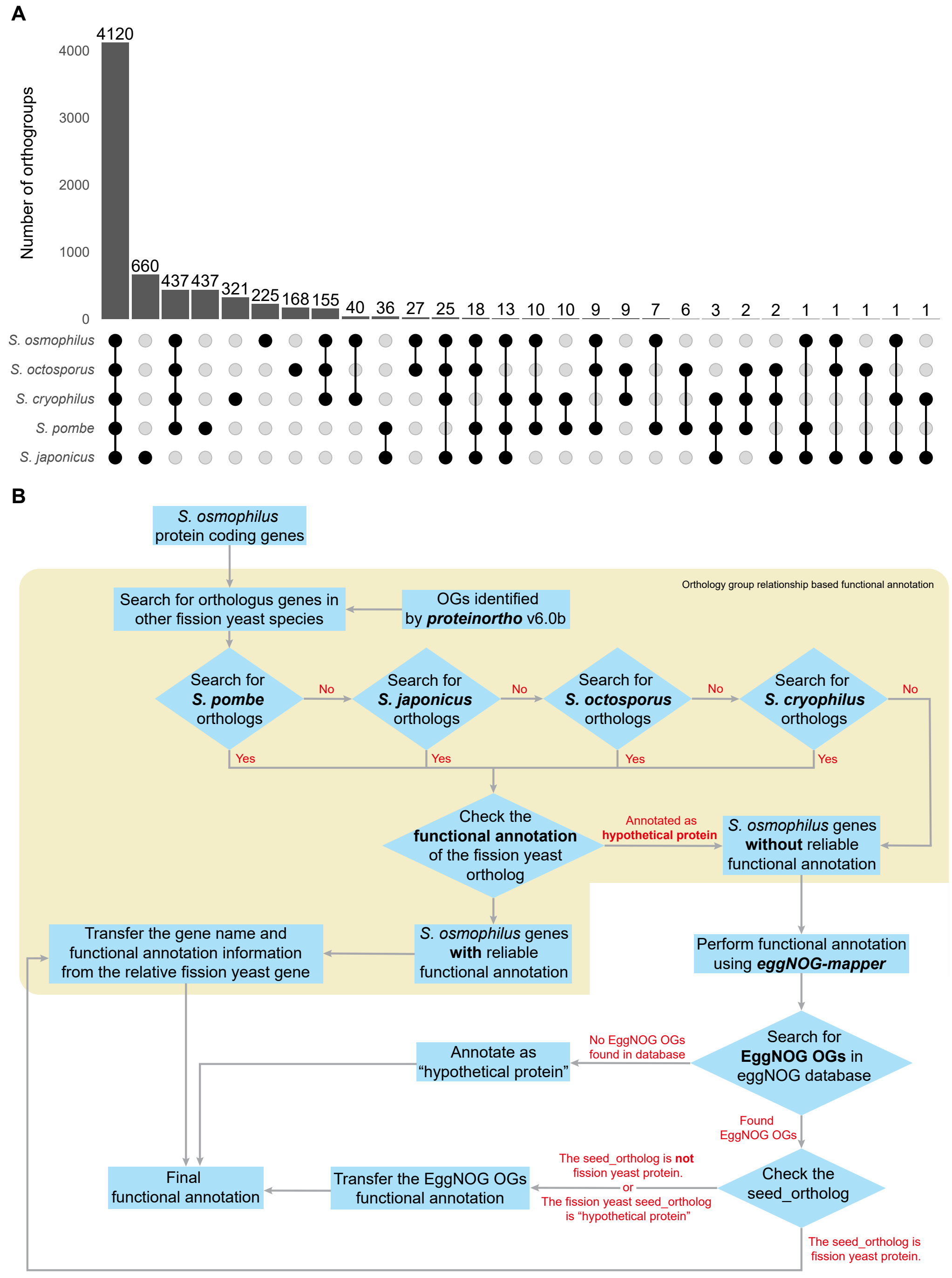

Figure S4. Orthogroup analysis and functional annotation of protein-coding genes.

(A) UpSet plot showing the numbers of species-specific orthogroups and orthogroups shared by more than one fission yeast species.

(B) Workflow of functionally annotating protein-coding genes in the nuclear genome.
