# Supplementary material for: A high-quality reference genome for the fission yeast Schizosaccharomyces osmophilus
Source: G3 (Bethesda). 2023 Feb 7;13(4):jkad028. doi: 10.1093/g3journal/jkad028 (PMC10085805; doi:10.1093/g3journal/jkad028)
Supplement: jkad028_Supplementary_Data [file jkad028_supplementary_data.zip › Figure_S5_G3-2022-403979.pdf]

Figure S5

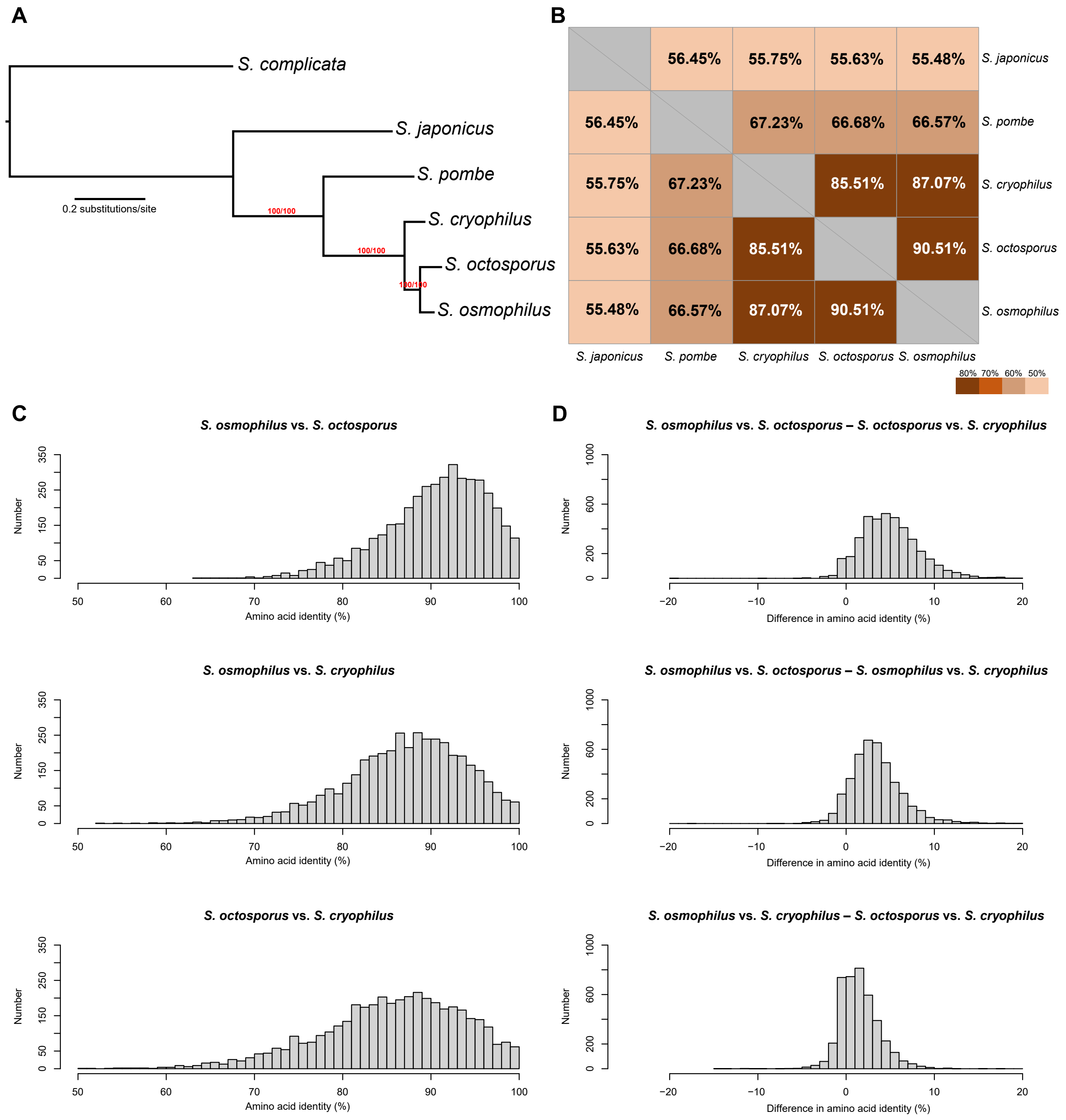

Figure S5. Species phylogeny analysis and evolutionary rate analysis.

(A) Maximum likelihood species tree constructed using the concatenation super-matrix of amino acid sequences of 1060 "complete and single-copy" BUSCO genes that present in all fission yeast species and the outgroup species *S. complicata*. The tree is rooted using *S. complicata*

as the outgroup. Branch labels are the SH-aLRT support value (%) and the UFBoot support value (%) calculated by IQ-TREE.

(B) Average amino acid percentage identities of fission yeast species pairs calculated using 4085 1:1:1:1:1 single-copy orthologs defined in this study. Pairwise amino acid percentage identities were calculated using Sequence Demarcation Tool Version 1.0 (Linux version). The heatmap was generated using the R package pheatmap (https://github.com/raivokolde/pheatmap).

(C) Histogram illustrating the distribution of pairwise amino acid identities of the 4085 single-copy orthologs.

(D) Histogram illustrating the distribution of differences in pairwise amino acid identities of the 4085 single-copy orthologs.
