# Supplementary material for: A high-quality reference genome for the fission yeast Schizosaccharomyces osmophilus
Source: G3 (Bethesda). 2023 Feb 7;13(4):jkad028. doi: 10.1093/g3journal/jkad028 (PMC10085805; doi:10.1093/g3journal/jkad028)
Supplement: jkad028_Supplementary_Data [file jkad028_supplementary_data.zip › Figure_S6_G3-2022-403979.pdf]

Figure S6

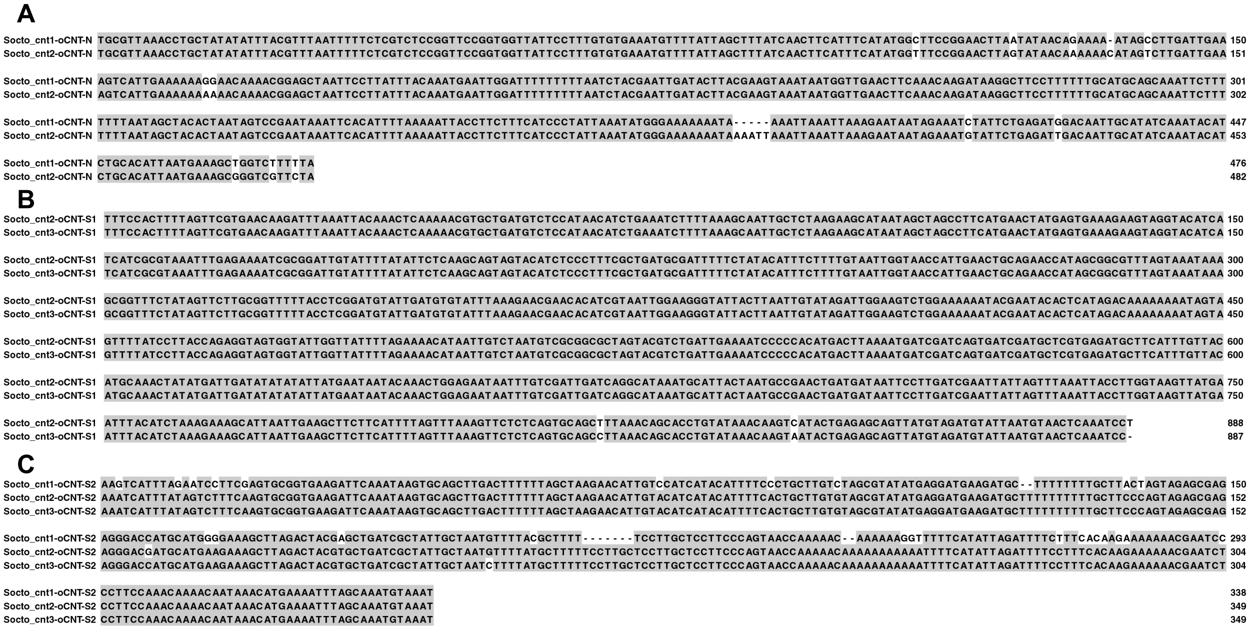

Figure S6. Sequence alignments of the oCNT-N, oCNT-S1, and oCNT-S2 repeats of *S. octosporus*. Nucleotides identical to the consensus are shaded in gray.

(A) Alignment of the nucleotide sequences of oCNT-N.

(B) Alignment of the nucleotide sequences of oCNT-S1.

(C) Alignment of the nucleotide sequences of oCNT-S2.
