# Supplementary material for: A high-quality reference genome for the fission yeast Schizosaccharomyces osmophilus
Source: G3 (Bethesda). 2023 Feb 7;13(4):jkad028. doi: 10.1093/g3journal/jkad028 (PMC10085805; doi:10.1093/g3journal/jkad028)
Supplement: jkad028_Supplementary_Data [file jkad028_supplementary_data.zip › Figure_S7_G3-2022-403979.pdf]

Figure S7

A

```
Sosmo_cnt1-CntA TGTCTTTACTGTGGATGATAATAGCAACCTTTACACTACGAAAAACATATAGTGCGTTTTTTTGTTTAATTGACAGTTCACACCACGGGCTAAGTAGGAAACCGTCTGAATCGCATGCAATGATATCGAAATAAACTAGCTCTTTTGAAC 150
Sosmo_cnt2-CntA TGTCTCAACTATGAATGAAAACAGCAACCTTTACACTACGAAAAACATATAGTGCGTTTTTTTGCTTAATTGACAGTTCACACCACGGGCTAAGTAGGAAACCGTCTGAATCGCATGCAATGATATCGAAATAAACTAGCTCTTTTGAAC 150

Sosmo_cnt1-CntA AGTATCAATAATCTGTGGTTTAGCCAAAATAAGGAGAAGAAAAATCGTTTGGCGCCCTTTTTTTAATTTATGCC 224
Sosmo_cnt2-CntA AGTATCAATAATCTGTGGTTTAGCCAAAATAAGGAGAAGAAAAATCGTTTGGCGCCCTTTTTTTAATTTATGCC 224
```

B

```
Sosmo_cnt1-CntB TACTAAAGAAGACTTCAATTCTCAAAATGTCCTTGGTTAAATGAATCAAGCTTGAGCGGATTGGCAAGTTCTTTTAGGTCACTGTTGTCAAAAAATACAAAAGATTATTCTTAAAGTGTCAAGGCGTTGGTATAATTATGATAAAAGCAT 150
Sosmo_cnt2-CntB TACTGGAGAAGACTTCAATTCTCAAAATGTCCTTGGTTAAATGAATCAAGCTTGAGCGGATTGGCAAGTTCTTTTAGGTCACTGTTGTCAAAAAATACAAAAGATTATTCTTAAAGTGTCAAGGCGTTGGTATAATTATGATAAAAGCAT 150
Sosmo_cnt3-CntB TACCAAAAAAGACCTCAATTCTCAAAATCTCCTTGGTTAAATGAATCAAGCTTGAGCGGATTGGCAAGTTCTTTTAGGTCACTGTTGTCAAAAAATACAAATGATTATTCTTAAAGTGTCAAGGCGTTGGTATAATTATGATAAAAGCAT 150

Sosmo_cnt1-CntB TATCAATATTTCAATCTACCAGAATAAGAATAACTAGATAGGTATGAAGACTAAGCTATGCAGGCCTTTCTTTAATGATTGCATGCAACAAACAAAGCGTGTTTGTCAAGCAAAGCTGAAAACATGTTGCAGCAGCTTAAAAGTTACTTT 300
Sosmo_cnt2-CntB TATCAATATTTCAATCTACCAGAATAAGAATAACTAGATAGGTATGAAGACTAAGCTATGCAGGCCTTTCTTTAATGATTGCATGCAACAAACAAAGCGTGTTTGTCAAGCAAAGCTGAAAACATGTTACAGCAGCTTAAAAGTTACTTC 300
Sosmo_cnt3-CntB TATCAATATTTCAATCTACCAGAATAAGAATAACTAGATAGGTATGAAGACTAAGCTATGCAGGCCTTTCTTTAATGATTGCATGCAACAAACAAAGCGTGTTTGTCAAGCAAAGCTGAAAACATGTTACAGCAGCTTAAAAGTTACTTC 300

Sosmo_cnt1-CntB GTGATTT-AAATAGTCGAGGGACATTATATATCGAAGGATAGGTAAAAAGAAAATCTTTATTGTCGACGGCTAGGAAGAAAGCATTATAATTATGGCTACGCATGAATGATAGCTTTATTTATTTATTTTAACATTAAAGCAGTGAACTG 449
Sosmo_cnt2-CntB GTGATTTAAAAAAGTCGAGGGACATTATATATCGAAGGATAGGTAAAAAGAAAATCTTTATTGTCGACGGCTAGGAAGAAAGCATTATAATTATGGCTACGCATGAATGATAGCTTTAATTATTTATTTTAACATTAAAGCAGTGAACTG 450
Sosmo_cnt3-CntB GTGATTTAAAAAAGTCGAGGGACATTATATATCGAAGGATAGGTAAAAAGAAAATCTTTATTGTCGACGGCTAGGAAGAAAGCATTATAATTATGGCTACGCATGAATGATAGCTTTAATTATTTATTTTAACATTAAAGCAGTGAACTG 450

Sosmo_cnt1-CntB AGCATCCACTGAAAGAATAAATTAAAAAACACAACTAGTAAATAAACAGGTGCTACCGTTTTTATATTTCAGCCAAGAATGATGTTCTGTCGGGGGTTTCTTTAGGAAGAGCTGGTGTCTTGCACTCTGAAGGTATTATGTGAAAGAATA 599
Sosmo_cnt2-CntB AGCATCCACTGAAAGAATAAATTAAAAAACACAACTAGTAAATAAACAGGTGCTACCGTTTTTATATTTCAGCCAAGAATGATGTTCTGTTGGGGGTTTCTTTAGGAAGAGCTGGTGTCTTGCACTCTGAAGGTATTATGTGAAAGAATA 600
Sosmo_cnt3-CntB AGCATCCACTGAAAGAATAAATTAAAAAACACAACTAGTAAATAAACAGGTGCTACCGTTTTTATATTTCAGCCAAGAATGATGTTCTGTTGGGGGTTTCTTTAGGAAGAGCTGGTGTCTTGCACTCTGAAGGTATTATGTGAAAGAATA 600

Sosmo_cnt1-CntB TTATTGTTTTACTGCTATCTCTTAAGGCTGT 630
Sosmo_cnt2-CntB TTAGTGTTTTACTGCTATCTCTCCAGGTTGT 631
Sosmo_cnt3-CntB TTAGTGTTTTACTGCTATCTCTCCAGGCTGT 631
```

C

```
Sosmo_cnt1-CntC GTGCCGCAAAAAAGACGTGTATAAAAAGTCAACGAAGTAATAGTAAAATTCATTATAGCATTTATTCATTAAGCAGCGATATCGTTTATGTGAAGGGTGCTATCGTTAACGTTTTTGAACTCACATATTCAAGGCATTTACTTCGGTCGT 150
Sosmo_cnt3-CntC GTGCCGCAAAAAAGACGTGTATAAAAAGCCAACGAAGTAATAGTAAAATTCATTATAGCATTTATTCATTAAGCAGCAATATCGTTTATGTGAAGGGTGCTATCGCTAACGTTTTTGAACTCACATATTCAAGGCATTTACTTCGGTCGT 150

Sosmo_cnt1-CntC CCTCTGTGATTATAAAATTTATCTCAGTCTGCTTTACTTAAATAAT-GAAAAGCAAAGAAAGGAACATTGGTTTTGAAGTCAAATGGCTTTCAGTTATCGTATTATTTACATTACTATGGGCAACGTCTACTTCAGATTTAGTGCGTACT 299
Sosmo_cnt3-CntC CCTCTGTGATTATAAAATTTATCTGAGTCTGCTCTACTTGAATAATGGAAAAGCAAAGAAAGGAACATTGGTTTTGAAGTCAAATGGCTTTCAGTTATCGTATTATTTACATTACTATGGGCAACGTCTACTTCAGATTTAGTGCGTACT 300

Sosmo_cnt1-CntC TTGATATGAGCTCGTTTTCATTTAGCATCATTAATACTATGCTAGCAAAGAAATAGGAAAAAAAACTTAGTTTAATGCTAGTCAGAATGATTACTCTGCTTCACTGAAGATTATTAAGGTATTTAGAAATAATATATATATATATATATA 449
Sosmo_cnt3-CntC TTGATATGAGCTCGTTTTCATTTAGCATCATTAATACTATGCTAGCAAAGAAATAGGAAAAAAAACTTAGTTTAATGCTAGTCAGAATGATTACTCTGCTTCACTGAAGATTATTAAGGTATTTAGAAATA--ATATATATATATATATA 448

Sosmo_cnt1-CntC TCTTTTTCAAATAACGTTCGTCAGATACTTTCTTAACTTTGCGTTTGTTTACTGAGAGAGCTGTACGATTTGAACACTTGGGTTAGGCTCGTGAGAGATAGTTCACTTCATGTTAAATAAATGCTGATGTGAAATGAAGTGACTAAAAAA 599
Sosmo_cnt3-CntC TCTTTTTCAAATAACGTTCGTCAGATACTTTCTTAACTTTGCGTTTGTTTACTGAGAGAGCTGTACGATTTGAACACTTGGGTTAGGCTCGTGAGAGATAGTTCACTTCATGTTAAATAAATGCTGATGTGAAATGAAGTGACTAAAAAA 598

Sosmo_cnt1-CntC ATAAAAGAAATTACAAATAACTAACAAACATAGACGCAATACGGATATCACGATCGTTTATTATTTCGTTGTCAGCTTCTTTCTTTTACGTAAAAAACTTTACTGAAAAATAAATTGCAAGCAGGATGGGCTGTACGTAACACGTCAAAC 749
Sosmo_cnt3-CntC ATAAAAGAAATTACAAATAACTAACAAACATAGACGCAATACGGATATCACGATCGTTTATTATTTCGTTGTCAGCTTCTTTCTTTTACGTAAAAAACTTTACTGAAAAATAAATTGCAAGCAGGATGGGCTGTACGTAACACGTCAAAC 748

Sosmo_cnt1-CntC CCTTTTGTTTTAATTTTCACTGTCTTGGCTGTCTTCACTGTAGCCTTTTACATGCAATTAACTAAAATAACTTTATATGAATAAACGTGGTATGTTCCGACACTCAAGTGGTGGTGTGGTACGGTGGGTGGGCTTAGCCCGGTTACTAAT 899
Sosmo_cnt3-CntC CCTTTTGTTTTAATTTTCACTGTCTTGGCTGTCTTCACTGTAGCCTTTTACATGCAATTAACTAAAATAACTTTATATGAATAAACGTGGTATGTTCCGACACTCAAGTGGTGGTGTGGTACGGTGGGTGGGCTTAGCCCGGTTACTAAT 898

Sosmo_cnt1-CntC ATTTCTTGTTCGACGGATGAGATAAAAAACATTGTTAAACTCATGTACGGAGCAATTGTATGGCTTATTTTGTTTATAGAGAAAGAAAGCACACATAATAGCATAGATTAATCCTAAATGCTTCCTTTGATCATAAGCTTTTCCATCTAT 1049
Sosmo_cnt3-CntC ATTTCTTGTTCGACGGATGAGATAAAAAACATTGTTCAACTCATGTACGGAGCAATTGTATGGCTTATTTTGTTTATAGAGAAAGAAAGCACACATAATAGCATAGATTAATCCTAAATGCTTCCTTAGATCATAAGCTTTTCCATTCAT 1048

Sosmo_cnt1-CntC GCGA 1053
Sosmo_cnt3-CntC GCGA 1052
```

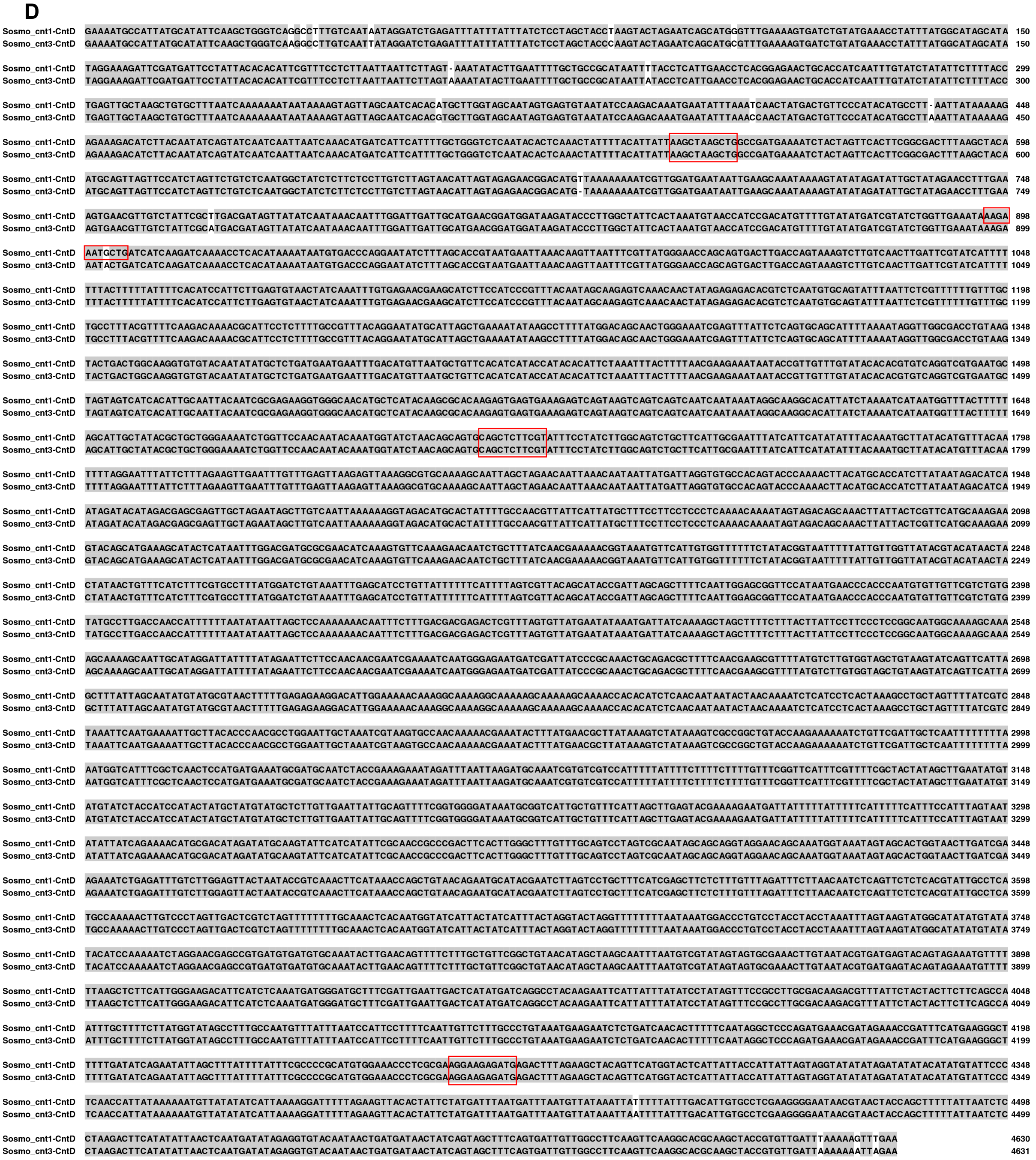

Figure S7: Sequence alignments of the *cnt* repeats of *S. osmophilus* and the occurrences of the 11-bp motif (boxed) in these repeats. Nucleotides identical to the consensus are shaded in gray.

(A) Alignment of the nucleotide sequences of CntA.

(B) Alignment of the nucleotide sequences of CntB.

(C) Alignment of the nucleotide sequences of CntC.

(D) Alignment of the nucleotide sequences of CntD.
