# Supplementary material for: A high-quality reference genome for the fission yeast Schizosaccharomyces osmophilus
Source: G3 (Bethesda). 2023 Feb 7;13(4):jkad028. doi: 10.1093/g3journal/jkad028 (PMC10085805; doi:10.1093/g3journal/jkad028)
Supplement: jkad028_Supplementary_Data [file jkad028_supplementary_data.zip › Figure_S8_G3-2022-403979.pdf]

Figure S8

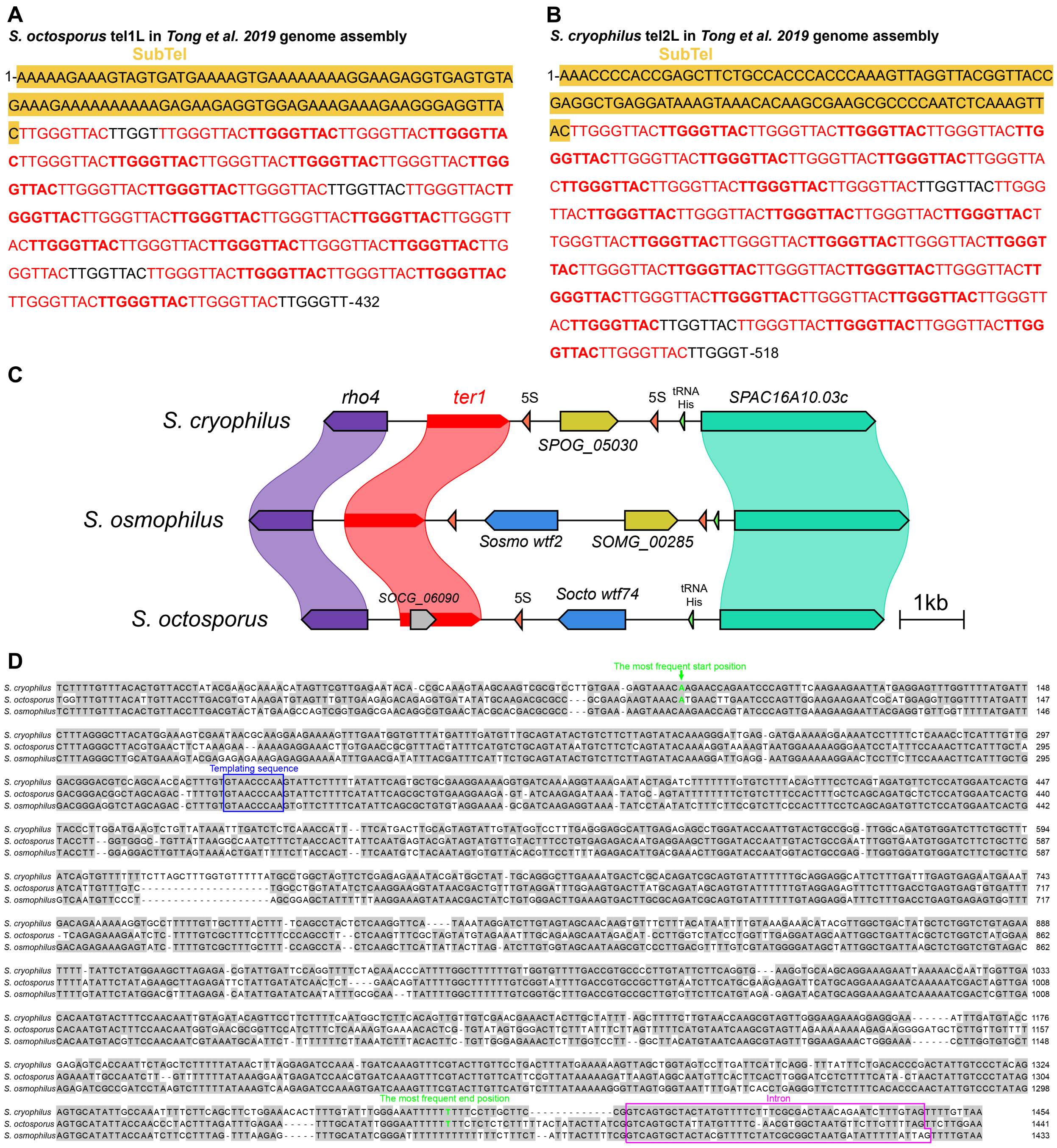

Figure S8. Comparative analysis of telomeric repeats and telomerase RNA genes in *S. osmophilus*, *S. octosporus*, and *S. cryophilus*.

(A and B) Sequence of a telomere in the PacBio sequencing-based *S. octosporus* genome assembly (A) and a telomere in the PacBio sequencing-based *S. cryophilus* genome assembly

(B) (Tong *et al.* 2019). The sequence juxtaposed to telomeric repeats are shown in a yellow background. The main type of telomeric repeats is shown in red and neighboring repeat units are distinguished by using regular and bold fonts alternately. All other types of repeat units are shown in black.

(C) Local synteny of the genomic region contain *ter1* and its flanking protein-coding genes. 5S rRNA genes and tRNA genes are labeled as red and green arrows respectively. Gene names shown are the names of the genes in *S. pombe* except for three species-specific genes (*SPOG_05030*, *SOMG_00285*, and *SOCG_06090*) and two *wtf* genes (*Sosmo wtf2* and *Socto wtf74*). The start and end positions of the *ter1* genes correspond to the most frequent start and end positions of mature *ter1* transcripts shown in D.

(D) Alignment of DNA sequences of the *ter1* gene and its flanking conserved sequences among *S. cryophilus*, *S. octosporus*, and *S. osmophilus*. Nucleotides identical to the consensus are shaded in gray. The templating region is indicated by a blue box. The most frequent start and end positions of mature *ter1* transcripts in *S. cryophilus* and *S. octosporus* reported in a previous study (Kannan *et al.* 2015) are highlighted in green letters. The introns (in a pink box) in the *ter1* genes of *S. cryophilus* and *S. octosporus* are as reported in a previous study (Kannan *et al.* 2015). The intron in the *ter1* gene of *S. osmophilus* are predicted based on sequence homology.
