# Supplementary material for: A high-quality reference genome for the fission yeast Schizosaccharomyces osmophilus
Source: G3 (Bethesda). 2023 Feb 7;13(4):jkad028. doi: 10.1093/g3journal/jkad028 (PMC10085805; doi:10.1093/g3journal/jkad028)
Supplement: jkad028_Supplementary_Data [file jkad028_supplementary_data.zip › Figure_S9_G3-2022-403979.pdf]

Figure S9

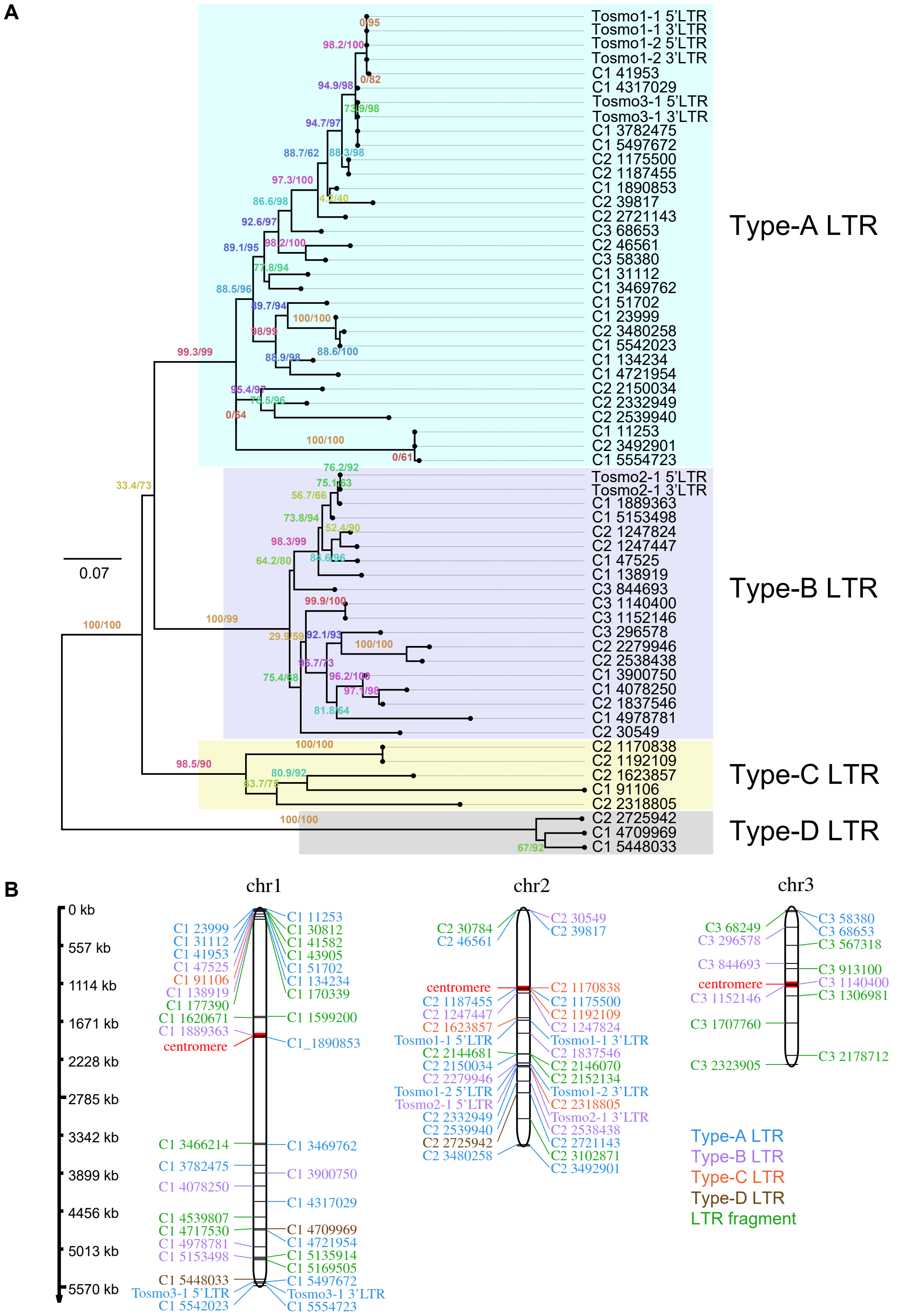

Figure S9. Phylogenetic relationship and chromosomal distribution of LTRs in *S. osmophilus*.

(A) Maximum likelihood tree of LTRs found in the *S. osmophilus* genome. The tree was rooted by midpoint rooting. Four major branches are denoted A, B, C, and D.

(B) Distribution of LTRs in the *S. osmophilus* genome.
